# Prevalence of unwanted pregnancy among Iranian women: an updated meta-analysis

**DOI:** 10.1186/s12884-019-2640-9

**Published:** 2019-12-11

**Authors:** Kourosh Sayehmiri, Fariba Ebtekar, Mozhdeh Zarei, Reza Ghanei Gheshlagh

**Affiliations:** 1 Prevention Center of Social-Mental Injuries, Ilam University of Medical Sciences, Ilam, Iran; 2 Department of Nursing, Faculty of Nursing and Midwifery, Kurdistan University of Medical Sciences, Sanandaj, Iran; 3 Department of Health in Emergencies and Disasters, Tehran University of Medical Sciences, Tehran, Iran; 4 Social Determinants of Health Research Center, Research Institute for Health Development, Kurdistan University of Medical Sciences, Sanandaj, Iran

**Keywords:** Unwanted pregnancy, Pregnancy, Meta-analysis, Iran

## Abstract

**Background:**

Unwanted pregnancy is a global issue with adverse outcomes for the mother, child, family, and society. Previous studies in Iran have reported different prevalence rates for unwanted pregnancy. This meta-analysis was aimed at estimating the overall prevalence of unwanted pregnancy among Iranian women.

**Methods:**

A total of 20 articles in English or Persian, published between 2012 and December 2018, were collected. The search was conducted in national and international databases, including Scientific Information Database (SID), MagIran, PubMed, Scopus, and Web of Science, using the following keywords: ‘Unplanned pregnancy’, ‘Unintended pregnancy’, ‘Unwanted pregnancy’, and ‘Mistimed pregnancy’. The data were analyzed using the meta-analysis method and the random effects model. Heterogeneity among studies was assessed using the I^2^ statistic. All analyses were performed using Stata, version 12.

**Results:**

Analysis of 20 studies with a total sample size of 16,298 showed that the prevalence of unwanted pregnancy among Iranian women was 26% (95% Confidence Interval [CI]: 23–28). This prevalence was higher in the regions 5 and 2 of Iran (27%) than the other regions, and had no significant decrease between 2012 and 2018 (*p* = 0. 937).

**Conclusion:**

More than one-fourth of pregnancies among Iranian women are unwanted. Providing training programs for couples who do not plan to have children along with the support policies aimed at stimulating population growth, can be an important step in overcoming the issue of unwanted pregnancy and reducing the illegal abortions related to it.

## Background

Unwanted pregnancy refers to a pregnancy that is mistimed or unwanted by one or both partners [1, 2].Of 210 million pregnancies that occur each year throughout the word, about 80 million (40%) are unwanted, and one in 10 women ends her pregnancy by an unsafe abortion [3]. Most of unwanted pregnancies occur in developing countries, and this problem increases the risk of mortality for both mother and child [4]. In Iran, 80,000 intentional abortions occur each year, mostly as a result of unwanted pregnancy [5].

Unwanted pregnancy can lead to increased stress, high risk behaviors, delay in prenatal care, and lack of desire to seek social support during pregnancy, therefore reducing the quality of life of women [1, 6]. Children born from unwanted pregnancy are more likely to be neglected by their parents and often have a poor relationship with their mother [5]. Due to the mother’s lack of interest in having a child, the risk of malnutrition, mortality, and mistreatment is higher in children born from unwanted pregnancy [7]. One of the complications of unwanted pregnancy is inadequate care or delay is receiving medical care during pregnancy that may lead to hypertension, preterm labor, low birth weight, negative feelings and feeling more pain during delivery due to experiencing unpleasant emotions during pregnancy, and depression before or after labor [7–9].

The actual prevalence of unwanted pregnancy in Iran may be higher than the reported rates, because in the Iranian society, some women tend to hide their unwanted pregnancy from others, and this makes it difficult to obtain accurate information on this issue [10]. Previous studies in Iran have reported different prevalence rates for unwanted pregnancy, varying from 13 to 42.3% [11, 12]. Given the fact that the management of unwanted pregnancy requires accurate and up-to-date information, the present study aims to estimate the prevalence of unwanted pregnancy among Iranian women.

## Methods

This is a systematic review and meta-analysis aimed at examining the prevalence of unwanted pregnancy among Iranian women, between 2012 and December 2018. Two previous meta-analyses conducted in Iran had reported the prevalence of unwanted pregnancy among Iranian women in 1998–2005 and 2000–2012 [2, 13]. We conducted extensive search in national and international databases, including Scientific Information Database (SID), MagIran, PubMed, Scopus, and Web of Science for studies published between 2012 and December 2018. Search terms used included ‘Unplanned pregnancy’, ‘Unintended pregnancy’, ‘Unwanted pregnancy’, and ‘Mistimed pregnancy’. In the Iranian databases, the search was conducted using the Persian equivalents of the keywords. The references of the articles were also reviewed to access more related articles.

## Study selection and data extraction

All observational studies published in Farsi and English reporting the prevalence of unwanted pregnancy in Iran were included in the analysis. In addition, unrelated, qualitative, review, interventional, and duplicated studies were excluded. Search for articles, selection of articles, quality evaluation, and data extraction were performed by two independent researchers, and disagreements between them would be resolved by the head of the research team who is experienced in meta-analysis. The following data were extracted for analysis: name of first author, publication year, setting of the study, language, sample size, and prevalence or frequency of unwanted pregnancy. The two reviewers independently evaluated the methodological quality of the studies using the Strengthening the Reporting of Observational Studies in Epidemiology (STROBE). This checklist has 22 items assessing 6 different aspects of each study, including title, abstract, introduction, methods, results, discussion, and financial support [14].

## Statistical analysis

The Stata software, version 12 was used for data analysis. The variance of unwanted pregnancy prevalence in each article was computed based on the binomial distribution formula by extracting the frequency sample size from published data. Heterogeneity was assessed using the Cochran’s Q test and the I^2^ statistic, and according to the values obtained, the random effects model was used to combine the studies and estimate the pooled prevalence. The heterogeneities of the studies were divided into; less than 25% (low heterogeneity), 25–75% (moderate heterogeneity) and more than 75% (high heterogeneity) [15]. We also analyzed the prevalence of unwanted pregnancy by region, article’s language, and methodological quality. Funnel plot based on the Egger’s test was used to examine publication bias. To identify heterogeneous causes, univariate meta-regression analyses was performed on the variables of the publication year and sample size.

## Results

**Fig. 1:**
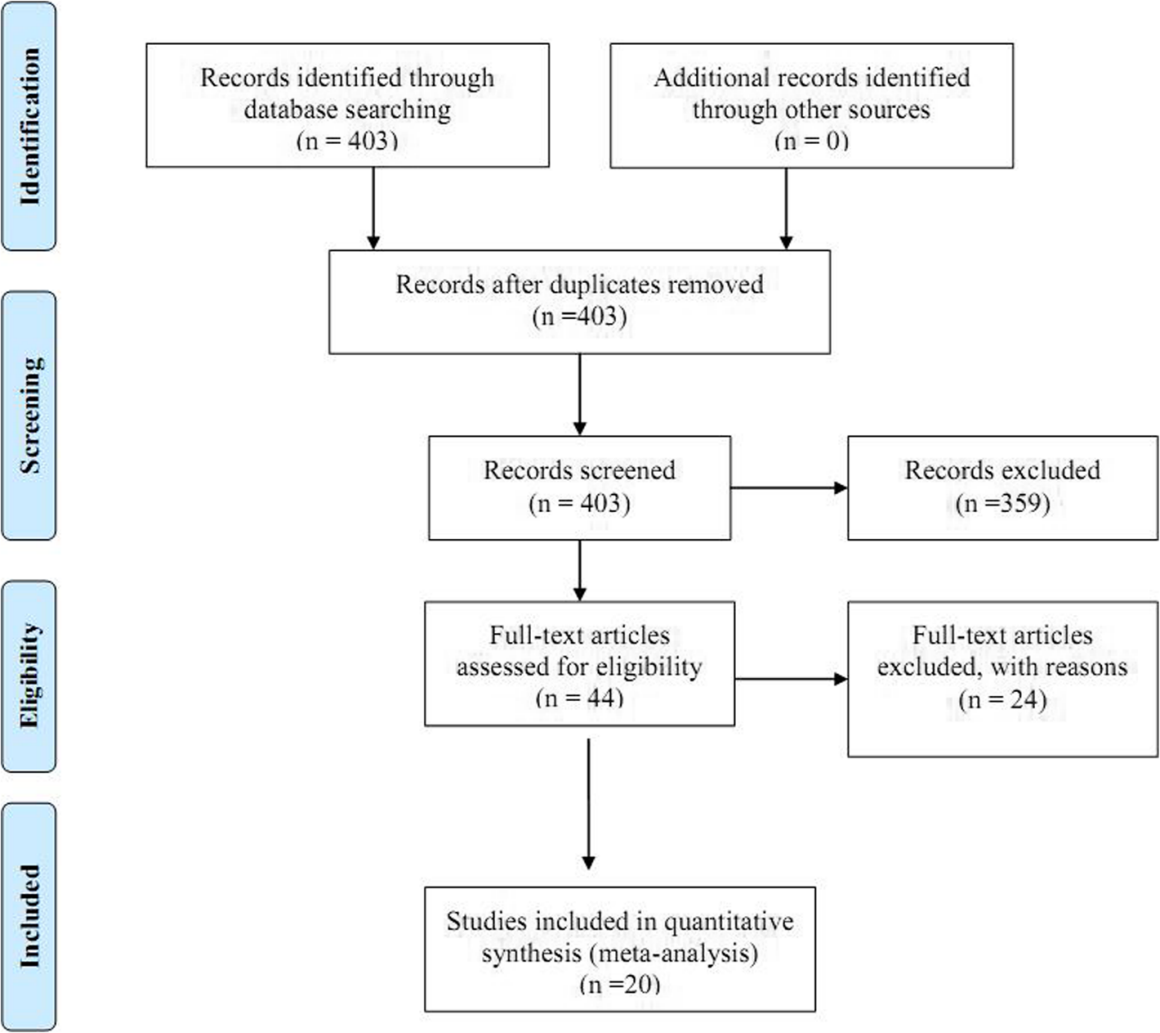
Process of selecting and screening articles based on the PRISMA statement

In the present systematic review and meta-analysis, all the studies conducted in Iran examining the prevalence of unwanted pregnancy among Iranian women were analyzed based on the Preferred Reporting Items for Systematic Reviews and Meta-Analyses (PRISMA) statement [16]. Two previous systematic reviews and meta-analyses had reported the prevalence of unwanted pregnancy in Iran in 1998–2005 and 2000–2012, therefore, we analyzed the articles published between 2012 and December 2018. In the primary search, 403 articles were found using the aforementioned keywords, of which 359 articles were excluded due to nonrelated topics. In the next step, the remaining 44 articles were examined, and 2 observational studies, 4 interventional studies, 2 systematic reviews, and 7 qualitative studies were excluded. One study was excluded because its full text was not available, and another one due to being conducted among women in temporary marriages. Finally, a total of 20 articles were included in the analysis (Fig. 1).

The total sample size was 16,298 (M = 815), and sample size varied from 100 to 5152. 7 articles were in Persian and 13 in English. In terms of methodological quality, 8 studies had a poor quality [17–24] and 12 had an average quality [5, 10–12, 25–32]. The description of the selected articles is presented in Table 1.

**Table 1:** Description of the selected articles

First Author	Year of Publication	Sample size	Place	Quality	Total Prevalence
Erfani [28]	2018	3000	Hamedan	Moderate	23
Omani-Samani [19]	2018	5152	Tehran	Low	19.8
Moeini [18]	2017	256	Bam	Low	28.1
Asadi Servestani [32]	2017	400	Shiraz	Moderate	17
Sajjadi [21]	2016	240	Izah	Low	35
Hossein-rashidi [26]	2016	518	Karaj	Moderate	31.6
Ebrahimzadeh [30]	2016	467	Khorramabad	Moderate	32.3
Shahbazin [10]	2015	248	Kangavar	Moderate	21.2
Valipour [22]	2015	220	Yazd	Low	28.6
Ashraf Ganjouei [31]	2015	231	Kerman	Moderate	30.3
Zaheri [24]	2015	1070	Sanandaj	Low	25.1
Ebrahimzadeh [29]	2015	887	Khorramabad	Moderate	25.3
Jarahi [5]	2014	300	Sarakhs	Moderate	21.7
Ostad norozi [25]	2014	1134	Sanandaj	Moderate	25.1
Pakdaman [12]	2014	305	Bandar Abbas	Moderate	42.3
Hassan-ghasemi [11]	2013	339	Gorgan	Moderate	13
Kiani [17]	2013	105	Mash-had	Low	29.5
Zare [23]	2012	1124	Shiraz	Low	24.3
Kefeshani [20]	2012	202	Lenjan	Low	17.9
Yazdani [27]	2012	100	Najafabad	Moderate	35

**Fig. 2:**
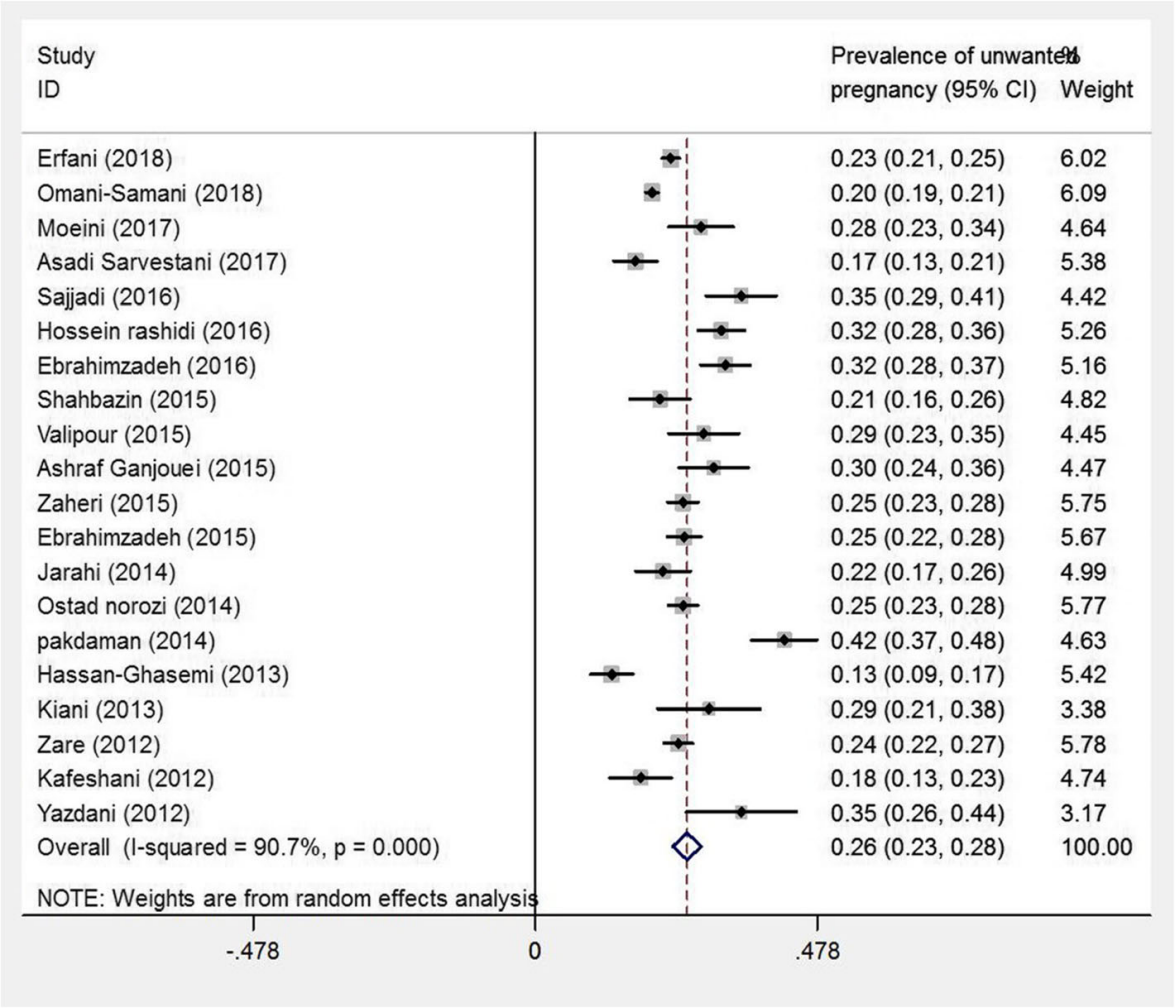
The diamond shown at the bottom of the figure indicates the weight of the squares. The horizontal diameter of the diamond shows the possible range of prevalence outcome. Two vertical lines are shown in the figure. The dotted vertical line which is in line with the diamond vertical axis shows the overall meta-analysis outcome (pooled prevalence). Prevalence of unwanted pregnancy and its 95% confidence interval among Iranian women based on name of first author and year of study using the random effects model. The middle point of each line segment shows the prevalence of unwanted pregnancy in each study, and the diamond shows the overall prevalence of unwanted pregnancy

The overall prevalence of unwanted pregnancy among Iranian women was 26% (95% Confidence Interval [CI]: 23–28) (Fig. 2). Subgroup analysis showed that the highest prevalence of unwanted pregnancy was in Iran’s region 2 [Esfahan, Fars, Bushehr, Hormozgan, Kohgiluyeh and Boyer-Ahmad, and Chaharmahal and Bakhtiari provinces (27% with 95% CI: 19–35)] and region 5 [Razavi Khorasan, North Khorasan, South Khorasan, Kerman, Yazd, and Sistan and Baluchestan provinces (27% with 95% CI: 24–31)].

**Fig. 3:**
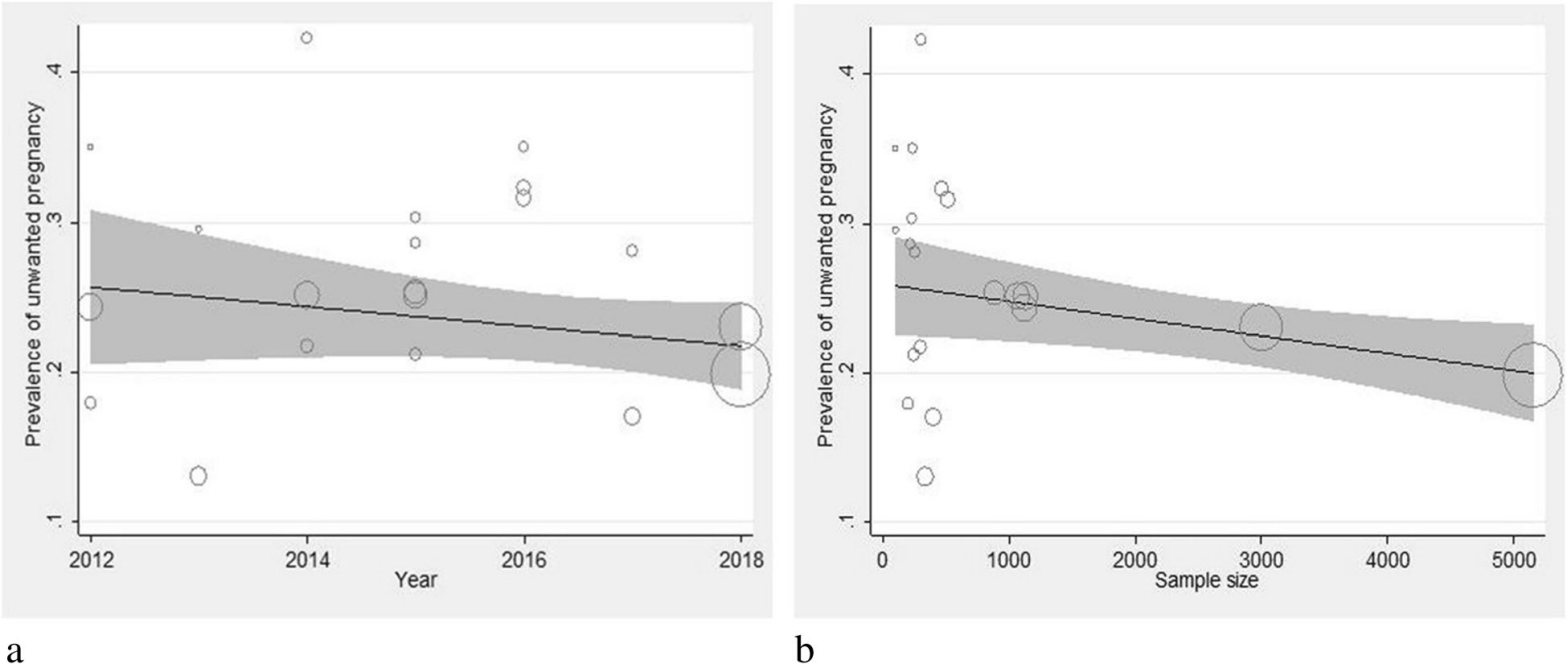
Meta-regression of the prevalence of unwanted pregnancy among Iranian women based on year of study (**a**) and sample size (**b**). Circles indicate the weight of studies

**Fig. 4:**
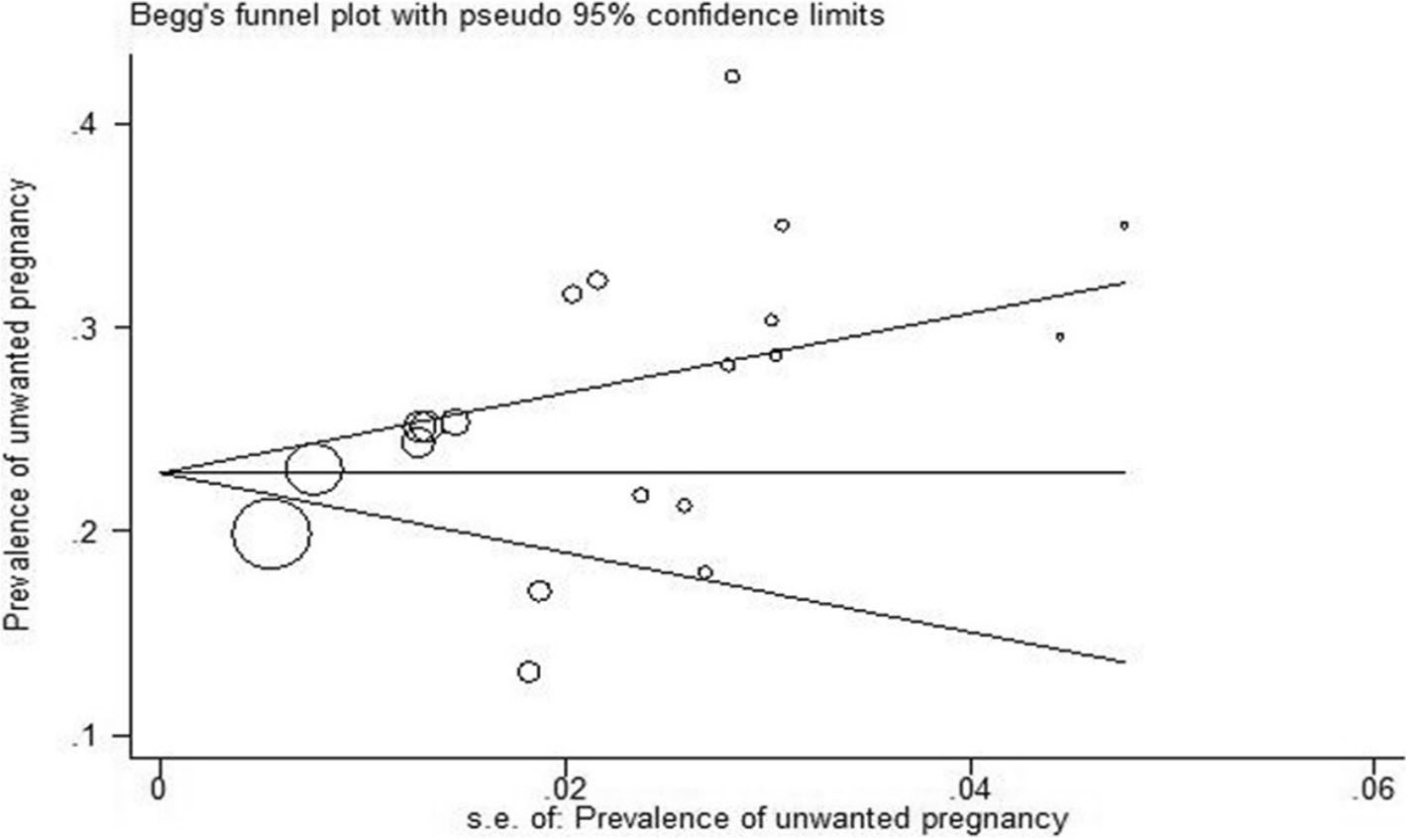
Publication bias

In terms of article’s language, prevalence of unwanted pregnancy was higher in the articles published in English (26% with 95% CI: 29–23) compared to those published in Persian (25% with 95% CI: 20–29). In addition, the prevalence of unwanted pregnancy was 26% (95% CI: 22–30) in the articles with an average methodological quality and 25% (95% CI: 22–29) in the articles with a poor methodological quality. The results of meta-regression analysis indicated no significant association between the prevalence of unwanted pregnancy and year of study (*p* = 0.937) and sample size (*p* = 0.234) (Fig. 3). According to the results, publication bias was statistically significant (*p* = 0.013). (Fig. 4).

## Discussion

The present study was aimed at estimating the overall prevalence of unwanted pregnancy among Iranian women. The results indicated that 26% of pregnancies in Iran were unwanted or unplanned. The prevalence of unwanted pregnancy has been found to be 27.1% in Ethiopia [3], 38.2% in Pakistan [4], and 47.3% in Turkey [33]. The results of a study by Finer showed that about half of pregnancies in the United States were unwanted [34]. Goto et al. (2002) also found a prevalence of 46.2% for unwanted pregnancy among Japanese women, and reported that about 40% of these women had experienced a previous unwanted pregnancy [35]. Unwanted pregnancy often has adverse physical and psychological consequences for both mother and child, and can deprive the mother of career and education opportunities and reduce the child’s wellbeing [2]. The lower prevalence of unwanted pregnancy among Iranian women compared to that in other countries could be attributed to cultural differences. In other words, unwanted pregnancy is viewed by Iranian women as a stigma, therefore, they are often reluctant to express or record it.

In the two previous meta-analyses conducted in Iran in 1998–2005 and 2000–2012, prevalence rates of 29.7 and 30.6%, respectively, were reported for unwanted pregnancy; these are higher than those found in our meta-analysis [2, 13]. In the present study, the results of meta-regression indicated no significant decreasing trend in the prevalence of unwanted pregnancy between 2012 and 2018. Regarding this finding, it is worth to note that due to population ageing, some policies have been adopted in Iran in recent years to increase birth rate and stimulate population growth, and family planning programs have been discontinued.

Lack of insertion of intrauterine devices (IUD), tubectomy, and vasectomy that are long-acting or permanent methods of birth control with a low failure rate, has led Iranian couples to use other birth control methods with high failure rates. Well-designed training programs are required to promote the use of these methods in Iran. Therefore, the policy to discontinue family planning programs, and the consequent lack of education for couples could be one of the reasons why the prevalence of unwanted pregnancy in Iran has remained relatively high in recent years. The removal of the family planning subject from students’ curricula could be another reason. A study on global and regional prevalence of unwanted pregnancy indicated that about 44% of global pregnancies in 2010–14 were unwanted. This study also showed that, compared to 1990–1994, the prevalence of unwanted pregnancy had a reduction by 30% in developed countries and by 16% in developing countries in 2010–14 [36].

Lack of reporting adequate information and lack of inclusion of gray literature in the analysis were two of the study limitations. Gray literature was not included in the meta-analysis because there is no comprehensive or specific database for it. Another limitation was the considerable difference between Iran’s provinces in the number of studies conducted; in some provinces, no study had ever been conducted on this issue, while other provinces had several studies on this topic. Another limitation was that the stratified sampling was not used in the primary selection of studies. These studies were not selected based on the population of provinces and cities, and population was not considered in combining the results of different studies. The weight assigned to each study in the random effects model was only based on sample size and standard deviation of the prevalence of unwanted pregnancy. Therefore, caution should be taken in generalizing the results to the whole country. One of the strengths of the study was that it was focused on a new topic. Therefore the study results can help the health authorities in Iran design healthcare programs and medical interventions aimed at addressing the issue of unwanted pregnancy among Iranian women.

## Conclusion

About one-fourth of pregnancies in Iran are unwanted, and unwanted pregnancy can have negative consequences for both mother and child. Unwanted pregnancy is a problem that seems not to be limited to a particular time or place, and is intensified by the use of traditional and unreliable birth control methods instead of evidence-based methods, therefore it often leads to illegal abortions. Through providing education for couples who have no plan to have children along with policies aimed at stimulating population growth, an important step can be taken to overcome the issue of unwanted pregnancy in Iran that imposes considerable costs and complications on families, the healthcare system, and the society as a whole, and reduce the mortality rate and maternal complications associated with illegal abortions following unwanted pregnancies.

## Data Availability

The datasets used and/or analysed during the current study are available from the corresponding author on reasonable request.

## Abbreviations

CI: Confidence Interval
IUD: Intrauterine devices
PRISMA: Preferred Reporting Items for Systematic Reviews and Meta-Analyses
SID: Scientific Information Database
STROBE: Strengthening the Reporting of Observational Studies in Epidemiology
